# Uncertainties on the prognostic value of admission D‐dimer on venous thromboembolism occurrence in patients with COVID‐19

**DOI:** 10.1002/rth2.12819

**Published:** 2022-11-22

**Authors:** Litao Zhang, Zhenlu Zhang

**Affiliations:** ^1^ Clinical Laboratory Wuhan Asia General Hospital affiliated to Wuhan University of Science and Technology Wuhan China; ^2^ Laboratory Medicine Wuhan Asia Heart Hospital Wuhan China

We read with great interest the paper by Woller and colleagues^1^ describing the predictive value of biomarkers, especially admission D‐dimer, on venous thromboembolism (VTE) occurrence in patients with coronavirus disease 2019 (COVID‐19) during hospitalization. These findings may provide useful evidence for better clinical use of these markers. However, there is an uncertainty that might influence the authors' conclusion that must be mentioned.

D‐dimer levels on admission would be greatly affected by the time from symptom onset to admission. For example (Figure 1), for a patient with COVID‐19 with a VTE diagnosis after admission, the D‐dimer level at admission may not be elevated if the patient is admitted in an early stage of COVID‐19 (Figure 1A); on the other hand, the D‐dimer level may be significantly elevated at the time of admission if there has been a long delay outside the hospital for any reason (Figure 1B). Therefore, there is great heterogeneity in the time from symptom onset to admission among individuals with COVID‐19 infection. In this regard, patients with negative D‐dimer on admission also should not be missed after hospital admission. Dynamic monitoring of D‐dimer might provide more predictive value in patients with COVID‐19. Of course, high‐quality studies are needed to validate.

**FIGURE 1:**
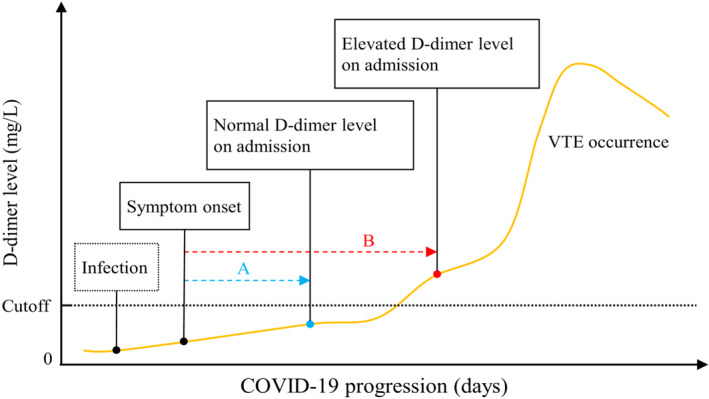
Dynamic changes of D‐dimers in patients with COVID‐19 with VTE occurrence. This figure shows a possible dynamic change of D‐dimers for patients with COVID‐19 with VTE occurrence during hospitalization. The admission D‐dimer would be affected by the time from symptom onset to admission (A with a normal D‐dimer on admission vs. B with an elevated D‐dimer on admission). COVID‐19, coronavirus disease 2019; VTE, venous thromboembolism

Actually, among the five studies included in the final analysis by Woller and colleagues,^1^ three provided the data about time from symptoms onset to admission, one of those included the time from symptom onset to admission in its multivariable analysis and concluded that from symptom onset, time to hospital admission was longer in patients with VTE in comparison with patients with no VTE.^2^ The time from symptom onset to admission is a crucial but neglected variable, which might cause potential uncertainty. Because the time from symptom onset to admission might differ greatly among individuals in real‐world experience and clinical studies. This might be attributed to the fact that the degrees of clinical symptoms differ greatly due to different host immune responses; furthermore, the time from symptom onset to the decision to visit the hospital varies for different patients; additionally, the medical resources and criteria for admitting patients with COVID‐19 are not comparable among different regions or institutions and so on.

The orders of D‐dimer testing have increased sharply since the outbreak of the COVID‐19 epidemic. Meanwhile, a number of studies about the various worse outcomes of patients with COVID‐19 were carried out. In conclusion, we should pay more attention to the role of the time from symptom onset to admission in related studies.

## AUTHOR CONTRIBUTIONS

LZ and ZZ drafted the manuscript.

## FUNDING INFORMATION

This work was supported by the Hubei Provincial Nature Science Foundation of China (2020CFB865) and the 2020 Wuhan Young & Middle‐Age Medical Backbone Training Programme.

## RELATIONSHIP DISCLOSURE

The authors declare that they have no conflicts of interest regarding this article.
